# An in-depth characterisation of European seabass intestinal segments for assessing the impact of an algae-based functional diet on intestinal health

**DOI:** 10.1038/s41598-023-38826-y

**Published:** 2023-07-19

**Authors:** Mariana Ferreira, Vera Sousa, Beatriz Oliveira, Ana Canadas-Sousa, H. Abreu, J. Dias, Viswanath Kiron, Luisa M. P. Valente

**Affiliations:** 1 CIIMAR/CIMAR-LA, Centro Interdisciplinar de Investigação Marinha e Ambiental, 4450-208 Matosinhos, Portugal; 2 ICBAS, Instituto de Ciências Biomédicas de Abel Salazar, Universidade Do Porto, 4050-313 Porto, Portugal; 3 EUVG, Escola Universitária Vasco da Gama, Quinta de S. Jorge, Estrada da Conraria, Castelo Viegas, 3040-714 Coimbra, Portugal; 4 ALGAplus, Production and Trading of Seaweed and Derived Products Ltd, 3830-196 Ílhavo, Portugal; 5 SPAROS Lda., 8700-221 Olhão, Portugal; 6 Faculty of Biosciences and Aquaculture, Nord University, 8049 Bodø, Norway

**Keywords:** Marine biology, Physiology

## Abstract

Sustainable farming of fish species depends on emerging new feed ingredients, which can alter the features of the digestive tract and influence animals’ overall health. Recent research has shown that functional feeds hold great potential for enhancing fish robustness by evoking appropriate responses at the intestine level. However, there is a lack of extensive and accurate descriptions of the morphology of the gastrointestinal tract of most farmed fish. We have characterised the intestine of European seabass thoroughly, by targeting four segments − anterior, mid, posterior and rectum. Results indicated that the anterior segment is mostly associated with absorption-related features; this segment has the largest absorptive area, the longest villi, and the highest number of neutral goblet cells (GC). The posterior segment and rectum have distinct histomorphometric features, but both seem to be important for immunity, displaying the highest count of acid GC and the highest expression of immune-related genes. The strongest proliferating cell nuclear antigen (PCNA) signal was observed in the anterior intestine and rectum, with PCNA^+^ cells appearing at the base of the villi and the corresponding villi branches. We have also evaluated the impact of a novel feed supplemented with a macro- and microalgae blend and found that there were no differences in terms of growth. However, the alterations observed in the mid intestine of fish fed the blend, such as thickening of the submucosa and lamina propria, an increased number of leucocytes, and higher expression of immune- and oxidative stress-related genes, suggest that algae may have an immunomodulatory effect. In the current article, we have described the morphology and expression patterns of the intestine segments of European seabass in detail and have presented a comprehensive report of the indices and methods used for the semi-quantitative and quantitative histomorphometric assessments, thereby providing useful information for future studies that aim to maintain intestinal health through dietary interventions.

## Introduction

Intestinal health indices are important for monitoring the welfare of farmed fish because some of the emerging new feed ingredients can alter the features of the digestive tract and have an effect on the overall health of animals^1^. The intestine is not only involved in the absorption of nutrients, but it concurrently acts as a physical and chemical barrier to prevent the passage of harmful compounds and microorganisms^2,3^. Nevertheless, extensive and accurate descriptions of the morphology of the gastrointestinal tract of farmed fish are few and far between^4,5^.

European seabass (*Dicentrarchus labrax*) is a carnivorous teleost fish of high economic relevance to the Mediterranean region^6^. However, intensification of its production associated with environmental stress factors and climate change often triggers unwanted diseases that severely affect the economic sustainability of the sector^7,8^. The concept of One-Health has prompted the aquaculture industry to search for ingredients capable of promoting fish health and welfare and allowed functional feeds to gain traction^9,10^. Macro- and microalgae are valuable sources of bioactive compounds that can act as immune modulators^11,12^. Hence, algae are promising candidates for functional aquafeeds, with potential to boost the immune responses of farmed fish^13,14^. To thoroughly understand the functionality of such products, their impact at the intestinal level has to be delineated because this organ is the key site where diet evokes local immune responses^1^.

Integrated approaches, by combining histomorphometric indices and molecular biomarkers, can provide valuable information about the effects of novel feed ingredients on the morphology and functions of the intestine of farmed fish^15–17^. It should be noted, however, that morphologic traits can differ among species and along their intestinal tract, and studies on intestinal features still rely on traditional and laborious methods that largely vary among studies. The available literature commonly identifies three main intestinal segments in fish: anterior (or proximal), mid, and posterior (or distal) intestine^4,5,18,19^. But since the fish intestine does not have easily distinguishable segments like those in mammals, there is currently no consensus on its morphological division and nomenclature^20^, resulting in difficulties when comparing results across studies. For example, in studies on European seabass, the definition of the anterior and posterior intestines varies. Some define the anterior intestine as the segment from the pyloric caeca to the ileorectal valve, and the posterior intestine as the portion after this valve, i.e. the rectum^21^. Others considered the posterior section and rectum as distinct portions of the posterior intestine^18^. Furthermore, many studies fail to provide an accurate description of the intestinal portions used in their analysis, and the lack of a standardised terminology only adds to the confusion.

Current histological parameters-based determination of the fish intestinal health depends on classical morphological changes, for example diet-induced adverse effects such as villi shortening. Many of the studies that have reported such morphological changes have relied on semi-qualitative analyses^22–24^, but quantitative approaches at the intestine level are becoming increasingly popular^18,25^. Moreover, a previous functional analysis based on transcriptomic profiling attributed specific functions to the segments of the European seabass intestine. This analysis reported that the anterior and mid sections are mostly associated with feed digestion and nutrient absorption, whereas the posterior/rectal segments are especially relevant for immune functions^26^. The posterior/rectal regions are also the preferred sections for understanding the diet-induced modulation of the microbial communities^27–30^. Nonetheless, the lack of a comprehensive study of the morphology and physiology of seabass intestine hampers the ability to selectively modulate specific targeted traits and hence contribute to precision nutrition.

We wanted to establish reference methods and guidelines for morphological studies at the intestine level, and we believe that this information can be used to assess European seabass intestinal health status, so that results can be easily compared with future studies. Hence, we performed an in-depth characterization of four segments of the seabass intestine (i.e., anterior, mid, posterior and rectum) using definable indices and following a holistic approach: (1) fast-track image analysis combined with traditional histology methods; and (2) evaluation of the gene expression patterns in relation to immunity, oxidative stress, and nutrient transport. These methods were then used to understand the impact of a functional diet supplemented with both micro- and macroalgae on the intestinal structure of European seabass. To analyse the histomorphometric traits, both quantitative and semi-quantitative assessments were performed to compare the performance of these two commonly used approaches for evaluating the intestinal health of fish.

## Materials and methods

### Ethical statement

Fish rearing and sampling were conducted using routine and animal husbandry practices of commercial farming operations. The fish trial and experimental protocols were approved by the Ethical Committee of Riasearch Lda. (Murtosa, Portugal) overseen by the National Veterinary Authority (DGAV, Portugal), in compliance with the guidelines of the European Union (Directive 2010/63/EU) and Portuguese law (Decreto-Lei no. 113/2013, de 7 de Agosto) on the protection of animals used for scientific purposes. The study is reported in accordance with the ARRIVE guidelines.

### Experimental diets, feeding trial and fish sampling

A commercial algae blend, which is composed of *Gracilaria* sp., *Nannochloropsis* sp. and *Aurantiochytrium* sp., was produced by the Portuguese company ALGAplus (Ílhavo, Portugal). Two isoproteic (51% dry matter, DM) and isoenergetic (23 kJ g^−1^ DM) diets were formulated and produced by Sparos, Lda. (Olhão, Portugal): a control diet – CTRL –, with moderate levels of fish meal (15%); and an experimental diet with 2% of the algae blend – Blend (Supplementary Figure S1).

The feeding trial was conducted in a saltwater recirculation system at the facilities of Riasearch Unipessoal Lda., Portugal. European seabass (initial body weight 118.6 ± 15.2 g) were randomly assigned to four 350 L fiberglass tanks (i.e., two tanks per dietary treatment) and fed the experimental diets for 8 weeks (54 days). Fish were hand-fed three times a day and at the end of every feeding cycle, apparent satiation of the fish was confirmed through visual observation. The system conditions were as follows: water temperature of 20 °C, salinity of 18‰, flow rate at 700 L/h (200%/h) and 12 h light/12 h dark photoperiod regime.

At the end of the feeding trial, and following a 48-h fasting period, 10 fish per tank were euthanised by anaesthetic overdose (MS222, 150 mg L^−1^). The different sections of the intestine were sampled, specifically the anterior region (i.e., right after the pyloric ceca), the mid intestine (i.e., the middle portion in between the anterior and posterior sections), the posterior region (i.e., right before the ileorectal valve), and the rectal section (i.e., right after the ileorectal valve). Samples were carefully washed and fixed in 4% formaldehyde (pH 7.0 ± 0.1) for histological evaluation or deep-frozen for gene expression analysis by RT-PCR.

### Histomorphological evaluation of the different intestinal segments of European seabass

**Figure 1:**
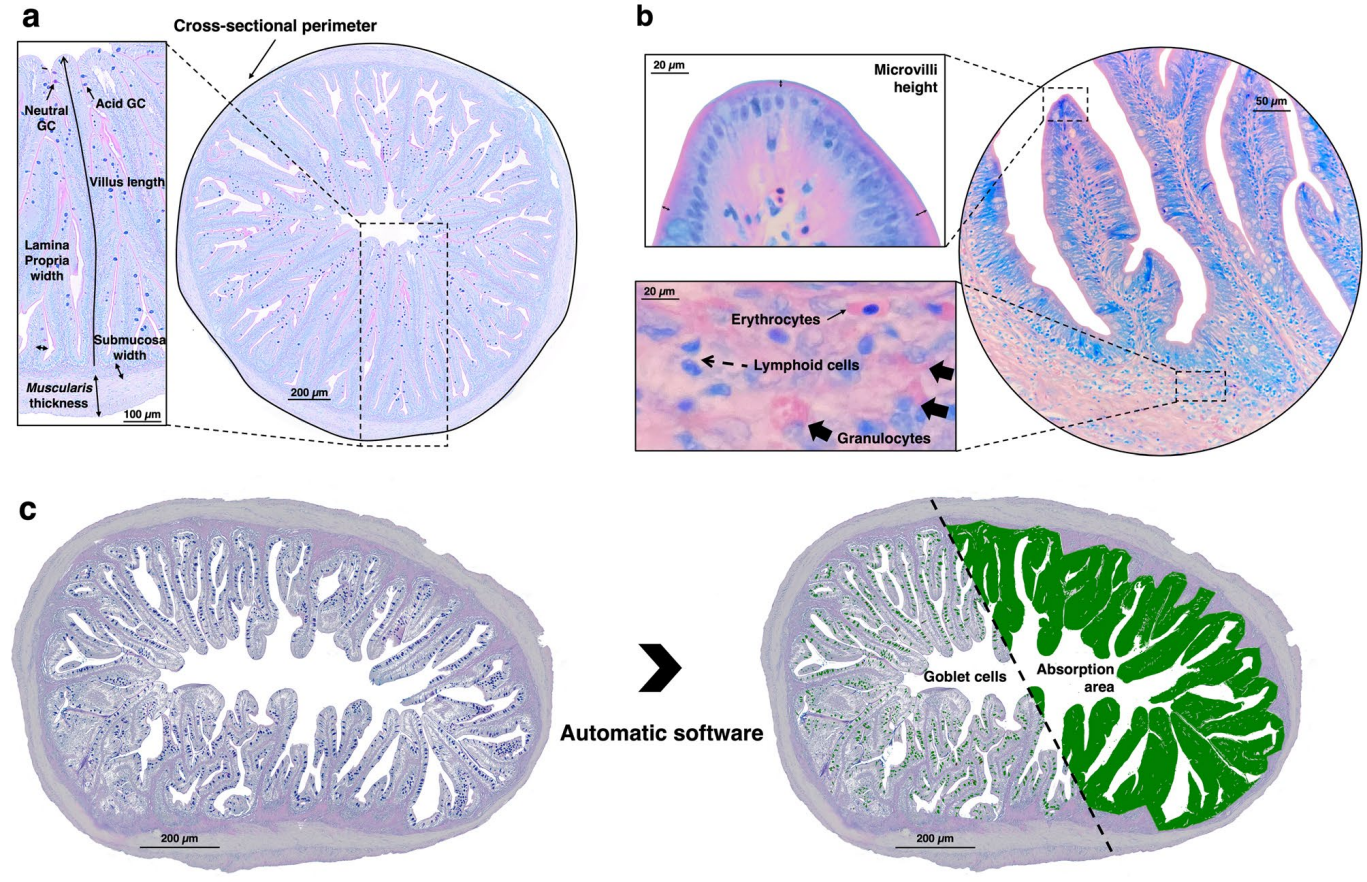
Strategy used for the quantitative analysis of the features of the intestine of European seabass. (**a**) Sections were stained with Alcian Blue/Periodic Acid Schiff (AB/PAS) to detect the acid and neutral goblet cells (GC), and measure the villi length, the submucosa and lamina propria width, the muscularis thickness and the cross-sectional perimeter. (**b**) Sections stained with May Grünwald-Giemsa (MGG) were used to measure the microvilli height, and to count the lymphoid cells and granulocytes in the submucosa and lamina propria. Note the morphology of the different cells found in submucosa and lamina propria: granulocytes are big, oval-shaped cells, with pink granules in the cytoplasm and a blue-stained nucleus in the periphery; lymphoid cells are small cells that stain blue; and erythrocytes are elongated cells with a pink cytoplasm and centrally located blue-stained nucleus. (**c**) The number of acid and neutral GCs and the absorption area (total area occupied by the villi) were automatically determined by the software. All measurements were performed using the imaging software Olympus cellSens. See Suplementary Table S1 for more details about the quantitative analysis.

After fixation, intestine samples were preserved in 70% ethanol. Samples were further processed according to standard histological procedures and embedded in paraffin. Five fish per tank were then selected for histological analyses (10 fish per dietary treatment). Intestinal cross-sections (3 μm) were obtained using a semi-automated rotary microtome (Leica RM 2245) and stained with Alcian Blue/Periodic Acid Schiff (AB/PAS; pH 2.5) and May Grünwald-Giemsa (MGG). Both staining techniques can be used to quantitatively measure almost all the evaluated parameters, except for the determination of the number of GC (AB/PAS specific) and measurement of microvilli height and leucocyte counting (MGG specific). Micrographs of each individual section were examined under a light microscope (Olympus BX51, Germany) with a camera (Olympus DP50). The sections (i.e., one cross-section per intestinal sample) were analysed using both quantitative (Fig. 1 and Supplementary Table S1) and semi-quantitative approaches (Table 1).

**Table 1:** Description of the scores used to evaluate the intestinal morphology of European seabass *–* semi-quantitative analysis.

Parameter	Score	Description
Villi length and integrity ^a^	1	Mucosal layer with a regular columnar epithelium with polarized and basally located nuclei, with no tissue damage. Anterior and rectum sections: long and thin organized villi. Mid and posterior sections: medium villi
	2	Typical villi length described in 1 for each section, and very few irregular/stubby folds (less than 1/4 of the section)
	3	Typical villi length, and few irregular/stubby folds (1/4 of the section)
	4	Reduction on the typical villi length, and few irregular/stubby folds (1/4 of the section)
	5	Reduction on the typical villi length, and some irregular/stubby folds (1/2 of the section)
	6	Short villi, and many irregular/stubby folds (1/2 to 3/4 of the section)
	7	Short villi, and most folds were irregular/stubby (3/4 of the section)
	8	Very short villi, and most folds were irregular/stubby (3/4 of the section) with tissue damage
	9	Very short and irregular villi, with apical displacement of nuclei and damaged tissue
Submucosa width ^b^	1	Mid, posterior and rectum sections: thin connective tissue between the base of the villi and the muscular layer. Anterior section: very thin submucosa layer
	2	Slightly wider connective tissue beneath very few villi (less than 1/4 of the section)
	3	Wider connective tissue beneath few villi (1/4 of the section)
	4	Wider connective tissue beneath some villi (1/2 of the section)
	5	Markedly wider connective tissue beneath some villi (1/2 of the section)
	6	Markedly wider tissue beneath many villi (1/2 to 3/4 of the section)
	7	Thick layer of the connective tissue beneath many villi (1/2 to 3/4 of the section)
	8	Thick layer of the connective tissue beneath most villi (more than 3/4 of the section)
	9	Extremely thick layer of connective tissue beneath all villi
Lamina propria width ^b^	1	Anterior, mid, and posterior sections: thin lamina propria and delicate core of connective tissue. Rectum: lamina propria is very thin and almost imperceptible
	2	Slightly wider lamina propria in very few villi (less than 1/4 of the section)
	3	Wider lamina propria in few villi (1/4 of the section)
	4	Lamina propria appears wider in some villi (1/2 of the section)
	5	Lamina propria was markedly wider in some villi (1/2 of the section)
	6	Lamina propria was markedly wider in many villi (1/2 to 3/4 of the section)
	7	Lamina propria is thick in many villi (1/2 to 3/4 of the section)
	8	Lamina propria is thick in most villi (more than 3/4 of the section)
	9	Lamina propria is very thick in all villi
Submucosa lymphoid cells	1	Anterior, mid, and posterior sections: lymphoid cells sparsely present in the submucosa. Rectum: monolayer of lymphoid cells appearing right beneath the villi, and lymphoid cells sparsely spread out through the rest of the submucosa
	2	Slight increase in the number of lymphoid cells in the submucosa compared to the normal appearance
	3	Anterior, mid, and posterior sections: lymphoid cells spread at a higher density (per area) throughout the submucosa. Rectum: few layers of lymphoid cells present right beneath the villi, and lymphoid cells sparsely spread out through the rest of the submucosa
	4	Marked increase in the number of lymphoid cells in the submucosa
	5	Submucosa thoroughly filled with lymphoid cells
Submucosa granulocytes	1	Granulocytes sparsely present in the submucosa
	2	Slight increase in the number of granulocytes in the submucosa
	3	Granulocytes spread at a higher density throughout the submucosa
	4	Marked increase in the number of granulocytes in the submucosa
	5	Submucosa heavily filled with granulocytes
Lamina propria lymphoid cells	1	Anterior, mid, and posterior sections: lymphoid cells sparsely present in the lamina propria and sometimes present right beneath the enterocytes. Rectum: almost no lymphoid cells visible in lamina propria
	2	Slight increase in the number of lymphoid cells in the lamina propria compared to the normal appearance
	3	Lymphoid cells spread throughout lamina propria at a higher density and intraepithelial lymphoid cells located at the base of the enterocytes
	4	Marked increase in the number of lymphoid cells in the lamina propria and intraepithelial lymphoid cells located at the base and/or apex of the enterocytes
	5	Lamina propria thoroughly filled with lymphoid cells and high density of intraepithelial lymphoid cells, located at the apex of the enterocytes
Lamina propria granulocytes	1	Anterior, mid, and posterior sections: granulocytes sparsely present in the lamina propria. Rectum: almost no granulocytes are visible in lamina propria
	2	Anterior, mid, and posterior sections: Slight increase in the number of granulocytes in the lamina propria compared to the normal appearance. Rectum: few granulocytes spread throughout the lamina propria
	3	Anterior, mid, and posterior sections: granulocytes spread throughout the lamina propria at a higher density (per area). Rectum: some granulocytes spread throughout all lamina propria
	4	Marked increase in the number of granulocytes in the lamina propria
	5	Lamina propria filled with granulocytes

^a^Adapted from Urán et al.^50^, Silva et al.^49^ and Penn et al.^48^; ^b^Adapted from Silva et al.^49^ and Penn et al.^48^.

A detailed description of the different histomorphological parameters evaluated quantitatively in the intestine of European seabass can be found in Supplementary Table S1. Briefly, two sections per sample were examined using an imaging software (Olympus cellSens Standard 2.2). The AB/PAS-stained section was used to determine the cross-sectional perimeter (CSP), the absorption area, the villi length, the muscularis thickness, the submucosa thickness, the lamina propria width and the number of goblet cells (GC), namely the acid and neutral GC (Fig. 1a). The ratios villi length/CSP, GC/absorption area, acid GC/absorption area, and neutral GC/absorption area were also calculated. On the other hand, the MGG-stained section was used to measure the microvilli height, and to manually count the number of lymphoid cells and granulocytes in the submucosa and lamina propria (Fig. 1b). As seen in Fig. 1c, the total number of acid and neutral GC and the absorptive area (total area occupied by the villi) were automatically determined using the analysis tools of the Olympus cellSens software.

An immunohistochemical procedure was used to determine the localization of the proliferating cells using a cell nuclear antigen (PCNA). Firstly, the sections were dewaxed and rehydrated. Then antigen was retrieved by immersing the slides in 0.01 M citrate buffer (pH 6.0), followed by irradiation on a microwave on high power (800 W) for 20 min. After cooling at room temperature, the slides were rinsed for 5 min in running tap water, and immunostained using the Novolink Polymer Detection System kit (RE7140-K, Leica Biosystems, Germany), according to the manufacturer’s instructions. Thereafter the sections were incubated overnight with the primary antibody − anti-PCNA mouse monoclonal antibody (Santa Cruz Biotechnology, EUA) in a humidified chamber, at 4 °C. The antibody was diluted 1:2000 in 1% bovine serum albumin (BSA)/phosphate buffered saline (PBS). Counterstaining was performed with haematoxylin. The area occupied by PCNA^+^ cells was automatically determined by the Olympus cellSens software, using the same procedures that were adopted for counting the GC (Fig. 1c and Supplementary Table S1).

Each MGG stained section was also semi-quantitively evaluated according to the criteria outlined in Table 1. To evaluate each intestinal segment's features, all samples were screened beforehand to ensure the accurate assignment of the score of 1, as morphologic traits vary throughout the tract. For example, in the anterior and rectum sections, a score of 1 indicates long, thin, well-organized villi with no tissue damage, while in the mid and posterior sections, it corresponds to medium-length villi with no tissue damage. Furthermore, as scores increase, so does the degree of alteration in comparison to score 1. This example demonstrates the importance of carefully assessing each section's specific morphologic traits to assign accurate and reproducible scores. Concerning the scores range, while villi length and integrity, submucosa thickness, and lamina propria width were assigned scores of 1–9, submucosa lymphoid cells, submucosa granulocytes, lamina propria lymphoid cells, and lamina propria granulocytes were assigned scores of 1–5. These scores were considered the most appropriate to define the different intrinsic intestinal segment's features.

### Expression of genes in the different intestinal sections of European seabass

Total RNA was extracted from the different intestinal sections (~ 30 mg; n = 16 per treatment), using TRIzol reagent (Invitrogen, USA) and NZY Total RNA Isolation kit (NZYTech, Portugal), according to the method described by Ferreira et al.^31^. The quantity and purity of the extracted RNA were spectrophotometrically evaluated, while RNA integrity was assessed using an agarose electrophoresis gel as previously described^17^. Using 1 μg of RNA, first strand cDNA was synthesized, and real-time (RT) PCR assays were performed on a CFX384 Touch Real-Time PCR Detection System (Bio-Rad Laboratories, USA) with SsoAdvanced Universal SYBR Green Supermix (Bio-Rad Laboratories, USA). Following the protocol described by Ferreira et al.^17^, reactions were performed in duplicate. The employed thermal cycling conditions were: 95 °C for 30 s, followed by 35 cycles of two steps of 95 °C for 5 s and 60 °C for 30 s. The specificity of the RT-PCR reaction was ensured by a post-amplification dissociation curve, while PCR efficiency for each gene was determined using a fivefold serial dilution of cDNA from all the samples used in the experiment. A total of 22 genes (four reference genes and 18 target genes) were analysed, including genes associated with a) immunity (i.e., i*gM*, immunoglobulin M; *cd4*, cluster of differentiation 4; *il-6*, interleukin-6; *il-8*, interleukin-8; *il-1β*, interleukin-1 beta; *tnf-α*, tumor necrosis factor-alpha; *tcrβ*, T-cell receptor antigen receptor beta chain; *tlr9*, toll-like receptor 9; *casp3*, caspase 3; *pcna*, proliferating cell nuclear antigen; *pisc1*, piscidine 1); b) oxidative stress (i.e., *gpx*, glutathione peroxidase; *sod*, superoxide dismutase; *cat*, catalase); and c) nutrient digestion and absorption (i.e., *alp*, alkaline phosphatase; *malt*, maltase; *fabp2*, intestinal fatty acid binding protein; *aqp1*, aquaporin 1). The primer sequences, accession numbers, annealing temperatures, and PCR efficiencies can be found in Supplementary Table S2. The stability of the reference genes (i.e., *18 s*, *β-actin*, *ef1α* and *gapdh*) was determined by the geNorm algorithm^32^, within the qbase + software, version 3.2 (Biogazelle, Zwijnaarde, Belgium—www.qbaseplus.com), and a normalization factor was calculated for each sample. The comparative critical threshold (ΔΔCT) method^33^ was used to calculate the relative abundance of the target genes, as described by Ferreira et al.^17^.

### Statistical analysis

The IBM SPSS software platform 27.0 (SPSS Inc., Chicago, IL, USA) was used to perform statistical analysis. The assumptions of normality and homogeneity of variances were checked by Kolmogorov–Smirnov and Levene's tests, respectively. Data were transformed when necessary. For comparisons between intestinal sections, one-way ANOVA followed by Tukey's multiple comparison test was performed. The level of significance used was *P* < 0.05. Independent samples t-test was applied to understand the differences between the two diets. A Spearmen's rank correlation coefficient test was applied to the histology-based variables of the quantitative and semi-quantitative data. Significant correlations were considered at the bilateral levels of 0.05 or 0.1. The principal component analysis (PCA) was performed using XLSTAT version 2022.1.2. (Addinsoft, USA) to differentiate the samples from the different study groups.

## Results

### Histomorphology of the different sections of the intestine of European seabass

**Figure 2:**
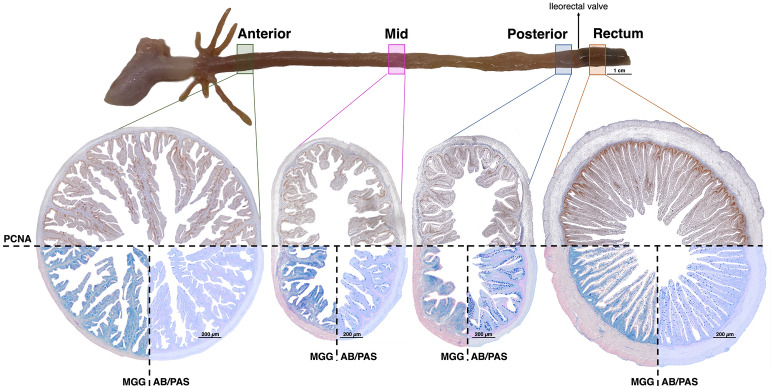
Histomorphology of the different sections of the intestine of European seabass. PCNA, Proliferating cell nuclear antigen (immunohistochemistry); MGG, May Grünwald-Giemsa (staining); AB/PAS, Alcian Blue/ Periodic Acid – Schiff (staining). Note the morphological differences as well as the location of the PCNA^+^ cells that characterize the distinct sections.

Quantitative analyses were performed to describe the micromorphology of the different intestinal sections. The results of the analyses are presented in Table 2 and the differences in the morphology of anterior, mid, posterior, and rectal sections are evidenced in Fig. 2.

**Table 2:** Histomorphology of the different intestine sections of European seabass using a quantitative analysis.

	Intestine sections			
	Anterior	Mid	Posterior	Rectum
Cross-sectional perimeter (CSP, mm)	14.4 ± 1.1 ^a^	11.0 ± 1.8 ^b^	10.3 ± 1.5 ^b^	13.6 ± 1.9 ^a^
Absorption area (mm^2^)	8.0 ± 1.1 ^a^	3.4 ± 0.9 ^b^	2.6 ± 0.9 ^c^	4.3 ± 1.1 ^b^
Muscularis thickness (μm)	112.8 ± 26.7 ^b^	135.2 ± 21.0 ^b^	128.3 ± 27.2 ^b^	263.8 ± 51.2 ^a^
Submucosa width (μm)	48.7 ± 12.1 ^b^	77.7 ± 20.9 ^a^	72.6 ± 23.6 ^a^	72.0 ± 11.8 ^a^
Lamina propria width (μm)	70.6 ± 10.7 ^a^	70.5 ± 16.1 ^a^	65.4 ± 12.0 ^a^	18.8 ± 6.1 ^b^
Villi length (μm)	1456.6 ± 160.3 ^a^	780.9 ± 118.0 ^bc^	577.8 ± 65.5 ^c^	900.7 ± 114.7 ^b^
Villi length/CSP (μm/mm)	101.5 ± 9.6 ^a^	72.3 ± 13.5 ^b^	56.6 ± 6.9 ^c^	67.2 ± 10.3 ^b^
Microvilli height (μm)	4.2 ± 0.6 ^b^	3.3 ± 0.4 ^c^	4.3 ± 1.0 ^b^	5.6 ± 0.7 ^a^
Goblet cells (GC)	2189.8 ± 601.5 ^b^	2415.7 ± 727.9 ^b^	3384.7 ± 974.4 ^a^	3700.9 ± 1325.6 ^a^
Acid GC	1883.3 ± 578.5 ^b^	2295.1 ± 775.0 ^b^	3273.3 ± 1036.5 ^a^	3451.3 ± 1252.0 ^a^
Neutral GC	306.5 ± 198.6 ^a^	120.6 ± 154.8 ^b^	111.4 ± 151.1 ^b^	249.6 ± 287.1 ^ab^
GC/absorption area (no. GC/mm^2^)	291.8 ± 88.2 ^c^	718.5 ± 153.5 ^b^	1287.8 ± 233.4 ^a^	869.1 ± 210.0 ^b^
Acid GC/absorption area (no. GCA/mm^2^)	249.3 ± 88.6 ^c^	682.2 ± 173.9 ^b^	1236.4 ± 236.8 ^a^	809.6 ± 193.0 ^b^
Neutral GC/absorption area (no. GCN/mm^2^)	42.6 ± 21.2	36.3 ± 47.2	51.4 ± 74.6	59.5 ± 63.3
Average area GC (μm^2^)	33.3 ± 7.2 ^b^	34.9 ± 6.3 ^b^	51.7 ± 10.9 ^a^	58.0 ± 10.7 ^a^
Submucosa lymphoid cells	65.6 ± 25.6 ^b^	85.9 ± 34.8 ^b^	66.9 ± 17.8 ^b^	187.8 ± 66.6 ^a^
Submucosa granulocytes	28.2 ± 12.2 ^a^	30.3 ± 9.9 ^a^	17.9 ± 6.0 ^b^	16.3 ± 4.8 ^b^
Lamina propria lymphoid cells	57.8 ± 24.6 ^a^	57.2 ± 23.2 ^a^	34.3 ± 9.6 ^b^	23.3 ± 8.4 ^b^
Lamina propria granulocytes	15.6 ± 5.8 ^a^	13.1 ± 4.8 ^a^	5.4 ± 1.5 ^b^	3.1 ± 1.3 ^b^
PCNA^+^ cells total area (mm^2^)	0.8 ± 0.2 ^a^	0.2 ± 0.1 ^c^	0.1 ± 0.1 ^c^	0.3 ± 0.1 ^b^
PCNA^+^ cells total area/absorption area (%)	7.4 ± 2.5 ^a^	5.3 ± 3.0 ^c^	5.6 ± 2.3 ^bc^	6.4 ± 2.5 ^ab^

Values presented as mean ± SD (n = 20 samples per section). One-way ANOVA followed by Tukey's multiple comparison test was performed. Different letters denote significant differences between intestinal sections. PCNA, Proliferating cell nuclear antigen. Figure 2 provides a visual representation of the characteristics of the distinct sections.

The anterior region had the largest absorptive area, the longest villi, the thinnest submucosa, and the largest total area of PCNA^+^ cells compared to all other sections. The posterior and rectal sections had higher GC and acid GC counts than the anterior and mid regions. The average area of the GC (including acid and neutral cells) was also significantly larger in these sections (i.e., posterior intestine and rectum). As for the GC and acid GC counts expressed per intestinal absorptive area, the highest values were for the posterior intestine, followed by the mid intestine and rectum, with the anterior intestine presenting the lowest value in comparison with all other sections. On the other hand, the anterior section had the highest count of neutral GC compared to the mid and posterior regions. However, when such neutral GC counts were expressed per absorptive area, we did not detect any significant differences between sections. The anterior and mid regions had significantly higher number of granulocytes in submucosa and lamina propria, and lymphoid cells in lamina propria compared to the posterior and rectal sections. The rectum had the thickest muscularis, the narrowest lamina propria, the longest microvilli, and the highest lymphoid cells counts in submucosa, in comparison with the other intestinal regions.

Concerning the PCNA^+^ cells per absorptive area, the anterior intestine presented the highest values, but was not significantly different from the rectum. Moreover, as observed in Fig. 2, the PCNA signal in the anterior portion was detected in the basal part of the complex folds and in the villi branching points, while in rectum the PCNA^+^ cells were concentrated at the basis of the villi.

A semi-quantitative analysis with well-defined and validated scoring system is described in Table 1 and was applied to the different intestinal sections in order to describe the intrinsic intestinal segment's features (Supplementary Table S3). Likewise, the quantitative analyses, the semi-quantitative approach confirmed that the anterior intestine is characterized by long villi with very few irregular folds and a thin submucosa layer. The mid and posterior segments had medium sized villi with few irregular folds, and the rectum had a very thin lamina propria with very few granulocytes. It is important to note that the attributed scores are specific to each section and cannot be used to compare differences between them.

### Gene expression patterns of the different sections of the intestine of European seabass

**Figure 3:**
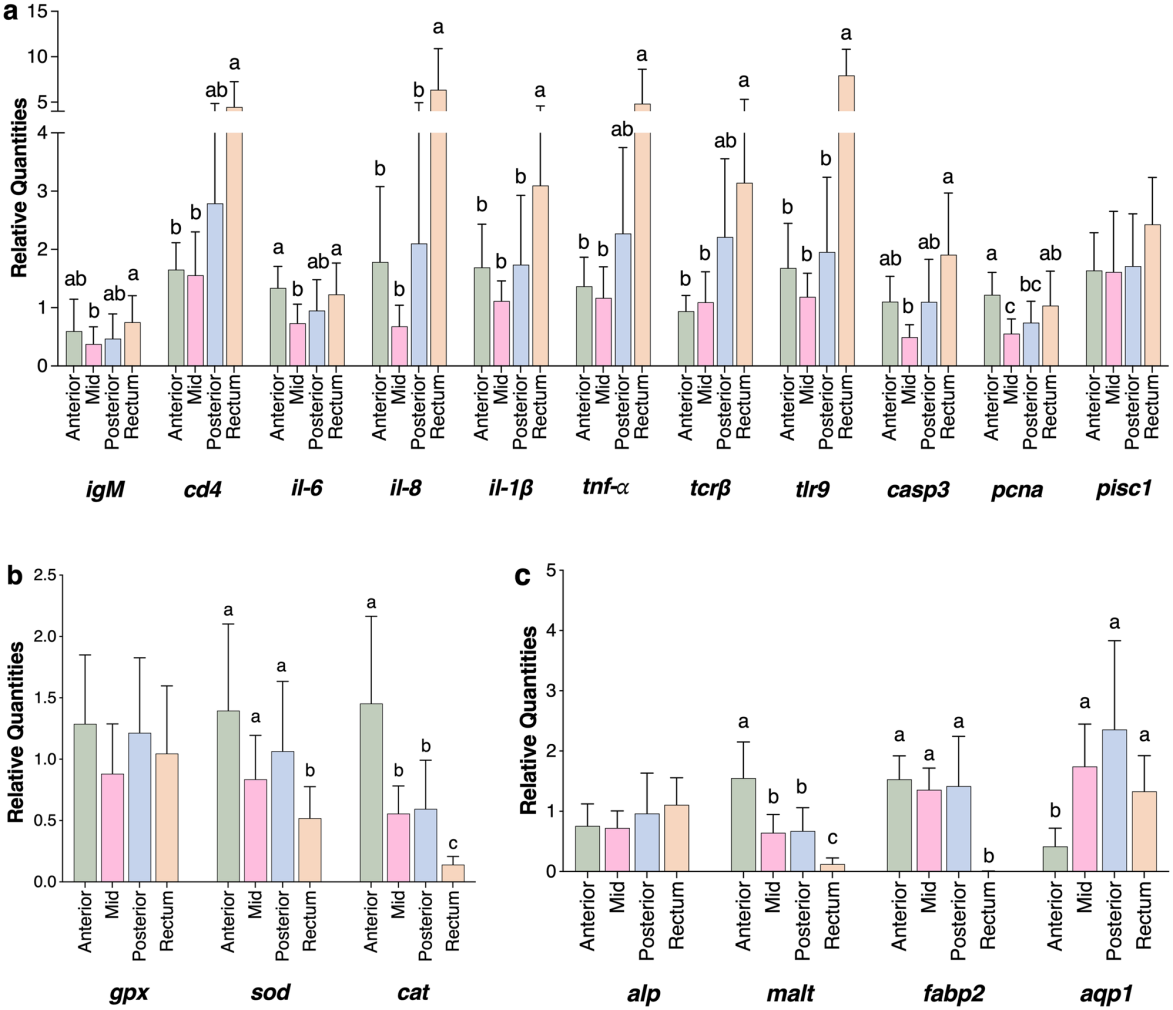
Gene expression in the different sections of the intestine of European seabass juveniles. (**a**) Immune-related genes; (**b**) genes associated with oxidative stress genes; and (**c**) genes related to nutrient digestion and absorption. Relative quantities in the intestine are presented as mean ± SD (n = 16 samples per section). One-way ANOVA followed by Tukey's multiple comparison test was performed, and different letters above the bars denote significant differences between intestinal sections.

A panel of 18 genes, involved in immunity, oxidative stress and nutrient digestion and absorption, were selected to determine the gene expression patterns associated with the different sections of the intestine of European seabass (Fig. 3). The expression of genes involved in the different immune pathways was, in general, higher in the rectum. Specifically, the mRNA levels of *il-8*, *il-1β* and *tlr*9 were significantly higher in the rectum compared to all other sections. The genes *cd4*, *tnf-α*, and *tcrβ* had higher expression in rectum only in comparison with the anterior and mid regions. In the rectum, the expression of *igM* and *casp3* was higher compared to only the mid intestine. The expression of *il-6* was higher in both the anterior and rectal section but compared to only the mid segment. In contrast, the *pcna* gene was found to have the highest transcript levels in the anterior region, followed by the rectum, with the mid region presenting the lowest expression values. There were no significant differences between the expression of the *pisc1* gene in the different intestinal sections (Fig. 3a).

The relative expression of genes associated with oxidative stress (i.e., *sod* and *cat*) differed significantly between the intestinal sections. The genes *sod* and *cat* were significantly downregulated in the rectum compared to all the other regions. The intestinal sections had similar levels of the mRNA of the gene *gpx* (Fig. 3b).

Concerning the genes related to nutrient digestion and absorption, the highest expression of the *malt* gene was observed in the anterior region, followed by the mid and posterior intestine, with the rectum presenting the lowest expression of this gene. While the *fabp2* gene was significantly lower in the rectum compared to all the other sections, the expression of *aqp1* was lower in the anterior region compared to the mid, posterior, and rectal sections. We did not observe any significant differences between the expression pattern of the *alp* gene along the intestinal tract (Fig. 3c).

### Principal component analysis – morphology and functionality of the different intestinal sections of European seabass

**Figure 4:**
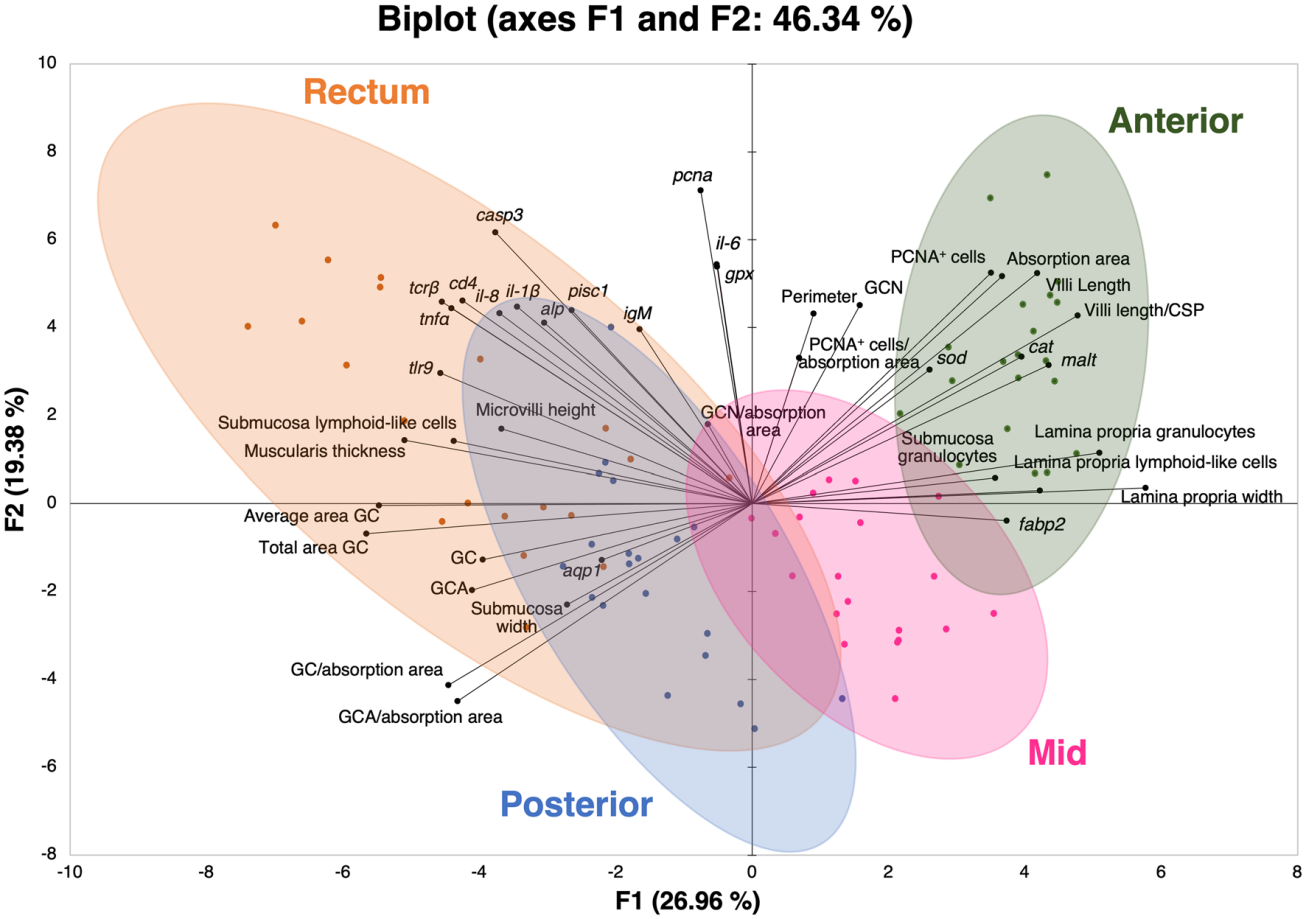
Principal component analysis (PCA) biplot for all variables analysed. Variables are displayed as loading vectors. Confidence ellipses (95%) are shown for each intestinal section: Anterior intestine – dark green; Mid intestine – pink; Posterior intestine – blue; and Rectum – orange.

Principal component analysis (PCA) was employed to visualise all the variables analysed in the anterior, mid, posterior, and rectal portions of the intestine of European seabass. The different intestinal sections were separated sequentially (as in the intestine) along the F1 axis, with the first two dimensions of the PCA biplot explaining 46.34% of the variability in the experimental data (Fig. 4). The variables absorptive area, villi length, expression of the genes *malt*, *fabp2*, *sod* and *cat*, cellular proliferation parameter (PCNA^+^ cells), granulocytes in submucosa and lamina propria, and lymphoid cells in lamina propria were found to be associated with the anterior intestinal section. The immune-related genes, muscularis thickness, microvilli height, acid GC, submucosa width, and expression of the genes *aqp1* and *alp* appeared in the quadrants where the posterior and rectum samples clustered in the PCA plot. The mid samples were in a different quadrant than the other sections (F1 positive/F2 negative).

### Growth performance of European seabass fed the experimental diets

**Figure 5:**
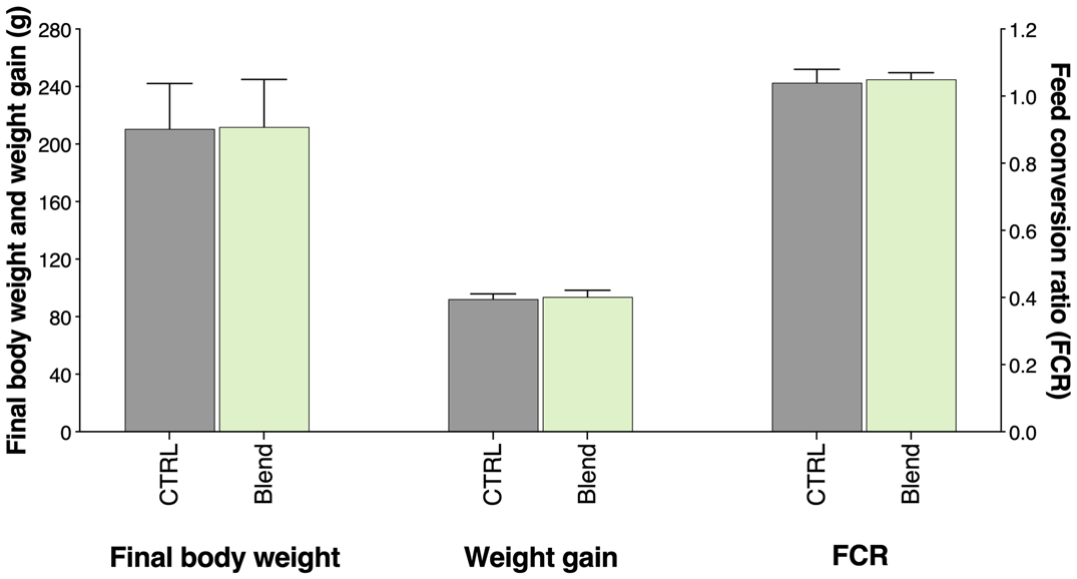
Growth performance of European seabass fed the control or the blend diet. Results are displayed as mean ± SD (n = 34 fish per treatment). FCR, feed conversion ratio.

After an 8-week feeding period, there was no significant difference in final body weight, weight gain, specific growth rate (SGR), and feed conversion ratio (FCR) of European seabass that were fed with a diet containing 2% of a commercial algae blend composed of *Gracilaria* sp., *Nannochloropsis* sp. and *Aurantiochytrium* sp. compared to those fed with a CTRL diet (Fig. 5).

### Impact of the algae blend-supplemented diet on the intestine of European seabass

To understand the impact of the algae blend-supplemented diet on the intestine of European seabass, both the histomorphology and gene expression patterns were analysed in the fish fed the CTRL and the Blend diets. Overall, the algae blend had very limited impacts on the morphology and functionality of the intestine, with most of the observed differences being located in the mid intestine (Table 3 and Supplementary Figure S2). A reduction in the villi length, number of GC and acid GC, and the total area occupied by the GC was observed in the mid intestine of fish fed the Blend diet compared to those fed the CTRL diet. The mid intestine of the fish fed the blend had thicker submucosa and wider lamina propria, both parts with a significantly higher number of lymphoid cells, and submucosa with more granulocytes. The area occupied by the PCNA^+^ cells per absorptive area in the mid intestine was also significantly increased after the blend diet feeding (Table 3). Alterations of the gene expression patterns were restricted to the mid region, with an upregulation of the *il-8*, *pisc1* and *gpx* genes in fish that consumed the algae-supplemented diet (Supplementary Figure S2). The differences observed in the other intestinal sections, in response to the blend diet, were limited to a higher number of lymphoid cells counts in the submucosa of the rectum, and higher number of granulocytes in the submucosa of the anterior and rectal sections (Table 3).

**Table 3:** Histomorphology of the different intestine sections of European seabass fed the control or the blend diets using a quantitative analysis.

	Anterior Intestine		Mid Intestine		Posterior Intestine		Rectum	
	CTRL	Blend	CTRL	Blend	CTRL	Blend	CTRL	Blend
Cross-sectional perimeter (CSP, mm)	14.3 ± 1.2	14.4 ± 1.0	11.4 ± 1.9	10.6 ± 1.5	10.6 ± 1.4	10.1 ± 1.7	13.7 ± 2.0	13.5 ± 2.0
Absorption area (mm^2^)	7.8 ± 1.1	8.2 ± 1.2	3.7 ± 1.0	3.1 ± 0.6	2.6 ± 0.6	2.7 ± 1.2	4.4 ± 1.1	4.09 ± 1.1
Villi length (μm)	1498.9 ± 184.3	1414.3 ± 127.8	837.1 ± 119.3 ^a^	724.6 ± 90.6 ^b^	580.6 ± 68.3	574.9 ± 66.1	939.8 ± 109.6	851.9 ± 107.7
Villi length/CSP (μm/mm)	104.9 ± 9.4	98.2 ± 9.0	74.8 ± 14.4	69.92 ± 12.9	55.1 ± 6.2	58.0 ± 7.6	69.7 ± 10.3	64.0 ± 9.9
Microvilli height (μm)	4.1 ± 0.5	4.2 ± 0.7	3.3 ± 0.5	3.3 ± 0.4	4.2 ± 0.9	4.5 ± 0.6	5.2 ± 0.8	5.9 ± 0.6
Muscularis thickness (μm)	101.8 ± 22.8	123.8 ± 26.8	128.1 ± 20.0	142.3 ± 20.6	117.5 ± 13.9	139.0 ± 33.4	253.9 ± 37.9	276.2 ± 65.0
Submucosa width (μm)	45.8 ± 5.0	51.6 ± 16.3	62.2 ± 12.4 ^b^	93.1 ± 15.5 ^a^	64.2 ± 9.3	81.0 ± 30.56	67.90 ± 12.54	77.14 ± 8.96
Lamina propria width (μm)	64.6 ± 9.0 ^b^	76.6 ± 9.0 ^a^	61.1 ± 8.2 ^b^	78.5 ± 17.3 ^a^	63.6 ± 14.6	67.3 ± 9.2	18.2 ± 5.4	19.5 ± 7.3
Goblet cells (GC)	2206.9 ± 652.8	2172.6 ± 580.6	2871.4 ± 705.7 ^a^	2010.6 ± 481.1 ^b^	3446.8 ± 1032.3	3322.7 ± 971.3	3656.8 ± 1395.5	3756.0 ± 1325.7
Acid GC	1901.9 ± 596.7	1864.6 ± 591.4	2810.9 ± 696.4 ^a^	1836.7 ± 523.9 ^b^	3347.0 ± 1040.3	3199.6 ± 1090.3	3475.4 ± 1386.4	3421.3 ± 1154.7
Neutral GC	305.0 ± 233.0	308.0 ± 170.3	60.5 ± 62.2	174.0 ± 194.2	99.8 ± 149.6	123.1 ± 160.6	181.4 ± 208.7	334.8 ± 359.4
GC/absorption area (no. GC/mm^2^)	306.7 ± 80.9	278.6 ± 97.0	789.5 ± 154.6	655.3 ± 129.1	1310.8 ± 221.4	1264.8 ± 256.1	827.9 ± 199.1	920.6 ± 225.1
Acid GC/absorption area (no. AGC/mm^2^)	260.8 ± 83.8	239.0 ± 96.5	775.1 ± 162.9 ^a^	599.6 ± 144.8 ^b^	1268.0 ± 199.1	1204.8 ± 278.0	784.2 ± 193.7	841.4 ± 200.5
Neutral GC/absorption area (no. NGC/mm^2^)	45.9 ± 24.1	39.6 ± 19.4	14.5 ± 10.4	55.7 ± 58.9	42.9 ± 71.1	60.0 ± 81.3	43.7 ± 46.8	79.3 ± 78.3
Average area GC (μm^2^)	30.7 ± 3.7	36.0 ± 9.0	36.3 ± 4.0	33.7 ± 7.8	54.5 ± 13.6	48.8 ± 6.9	58.1 ± 8.6	58.0 ± 13.5
Total area GC (mm^2^)	0.07 ± 0.02	0.08 ± 0.02	0.10 ± 0.02 ^a^	0.07 ± 0.02 ^b^	0.19 ± 0.08	0.16 ± 0.04	0.21 ± 0.07	0.20 ± 0.05
Submucosa lymphoid cells	64.5 ± 8.6	66.7 ± 8.0	69.4 ± 9.5 ^b^	102.4 ± 10.3 ^a^	67.7 ± 6.6	66.1 ± 4.8	177.1 ± 19.4 ^b^	224.2 ± 32.7 ^a^
Submucosa granulocytes	23.7 ± 2.9 ^b^	36.6 ± 5.2 ^a^	26.0 ± 2.4 ^b^	37.6 ± 3.9 ^a^	15.2 ± 2.1 ^b^	20.5 ± 1.3 ^a^	13.6 ± 1.2 ^b^	19.2 ± 1.4 ^a^
Lamina propria lymphoid cells	61.1 ± 7.7	54.5 ± 8.1	45.9 ± 4.6 ^b^	68.5 ± 8.0 ^a^	33.9 ± 2.7	34.6 ± 3.5	21.9 ± 2.7	24.7 ± 2.8
Lamina propria granulocytes	14.8 ± 1.7	16.34 ± 2.0	13.5 ± 1.4	15.8 ± 2.6	5.5 ± 0.4	5.7 ± 0.7	3.4 ± 0.6	3.7 ± 0.6
PCNA^+^ cells total area (mm^2^)	0.6 ± 0.2	0.6 ± 0.2	0.1 ± 0.1	0.2 ± 0.1	0.1 ± 0.1	0.2 ± 0.1	0.3 ± 0.1	0.3 ± 0.1
PCNA^+^ cells total area/absorption area (%)	8.0 ± 2.5	7.0 ± 2.5	3.7 ± 0.8 ^b^	7.1 ± 3.6 ^a^	5.5 ± 2.9	5.8 ± 1.5	6.1 ± 3.0	6.9 ± 1.8

CTRL – fish fed the control diet, Blend – fish fed the blend diet. Values presented as mean ± SD (n = 10 samples per dietary treatment), based on quantitative analysis. Independent samples t-test was employed to understand the significant differences between the study groups. Different letters denote significant differences between dietary treatments within each intestinal section. PCNA, Proliferating cell nuclear antigen.

**Figure 6:**
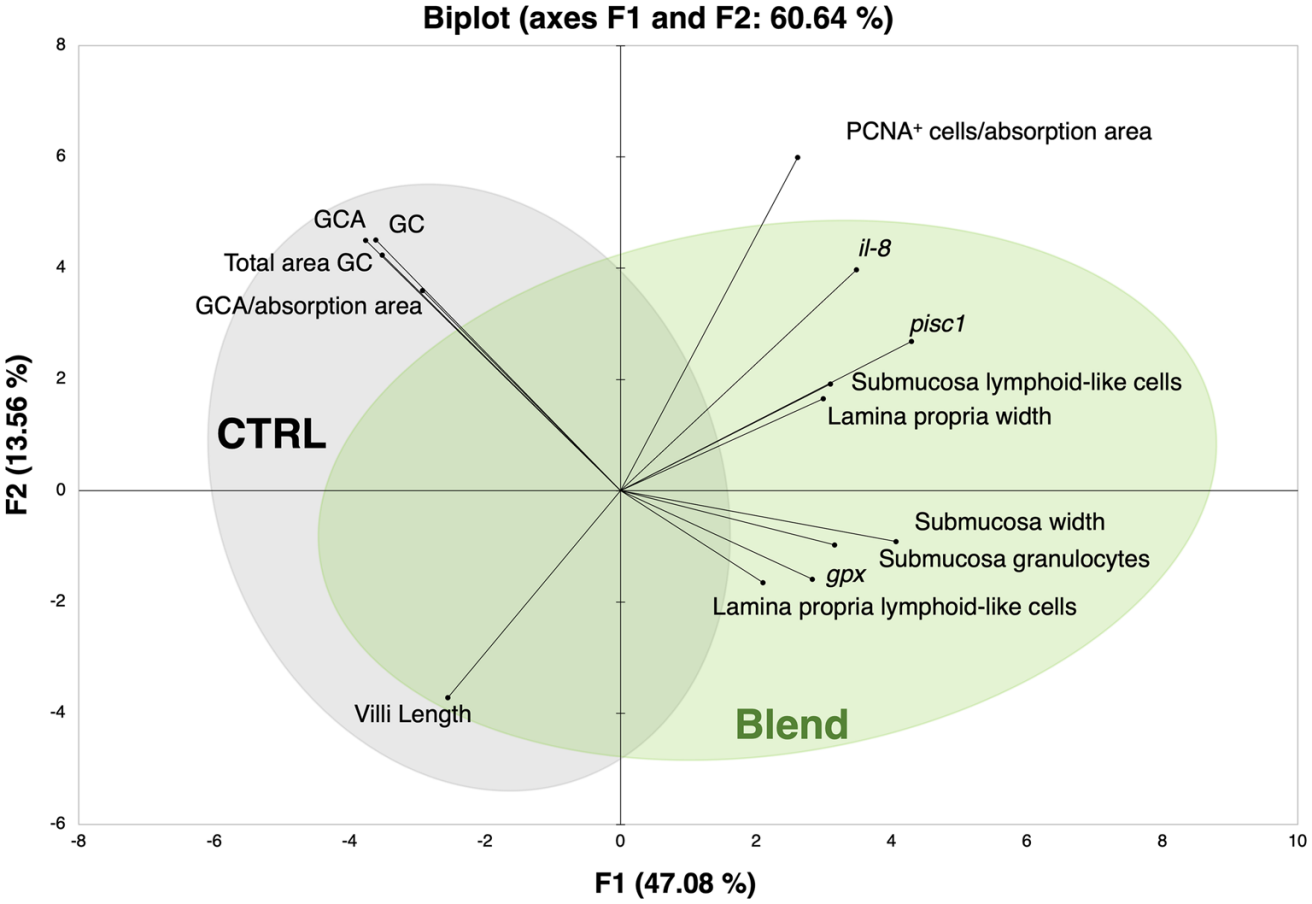
Principal component analysis (PCA) biplot for the variables that displayed significant differences in the mid intestine. Variables are displayed as loading vectors. Confidence ellipses (95%) are shown for each dietary treatment: CTRL – grey; Blend – green.

A PCA biplot created using all the analysed variables in each intestinal section could not discriminate between the CTRL and Blend samples (Supplementary Figure S3). On the other hand, when only the variables significantly affected by the experimental diet in the mid intestine were selected and visualised in a PCA biplot, the CTRL and Blend samples were separated along the F1 axis, with the first two dimensions explaining 60.64% of the variability of the data (Fig. 6). The blend samples were associated with immune-related parameters such as expression of *il-8* and the antimicrobial peptide *pisc1*, the presence of the lymphoid cells in submucosa and lamina propria, and the presence of granulocytes in submucosa. The lamina propria width and submucosa thickness and the oxidative stress-related gene *gpx* were also found in the F1 positive quadrant, where the blend samples were mostly clustered. The variables villi length, GC, acid GC, acid GC per absorptive area and total area occupied by the GC were positioned on the F1 negative quadrant and seemed to be associated with the control samples.

### Quantitative versus semi-quantitative approaches for evaluation of the intestinal health

The experimental diet-induced alterations of the intestine histomorphology were evaluated both quantitatively (Table 3) and semi-quantitatively (Table 4). In general, the differences captured by the quantitative assessment were also reflected in the semi-quantitative approach-based results, namely the increase in the submucosa thickness and lamina propria width, higher number of lymphoid cells in the submucosa and lamina propria, and granulocytes in the submucosa of the mid intestine of blend-fed fish. But some differences detected through the quantitative approach could not be perceived by the semi-quantitative analysis, namely the effects of the blend-diet on the villi length in the mid intestine, the increase in the lamina propria width in the anterior intestine and the rise in the number of granulocytes in the submucosa of the posterior intestine. Nevertheless, we observed a significant correlation between the variables of all the intestinal sections analysed quantitatively and semi-quantitatively, i.e., villi length, submucosa thickness, and lamina propria width, and presence of leucocytes in the submucosa and lamina propria (Supplementary Figure S4).

**Table 4:** Histomorphology of the different intestine sections of European seabass fed the control or the blend diets using a semi-quantitative analysis.

	Anterior intestine		Mid intestine		Posterior intestine		Rectum	
	CTRL	Blend	CTRL	Blend	CTRL	Blend	CTRL	Blend
Villi length and integrity	1.9 ± 1.3	3.2 ± 2.0	2.4 ± 1.4	4.4 ± 2.5	2.1 ± 1.3	2.6 ± 1.2	1.4 ± 0.7	2.2 ± 1.2
Submucosa width	1.4 ± 0.5	2.0 ± 1.3	2.4 ± 1.0 ^b^	4.3 ± 2.1 ^a^	2.2 ± 0.7	3.1 ± 2.2	1.8 ± 0.8	2.4 ± 0.5
Lamina propria width	1.9 ± 0.9	3.2 ± 2.1	1.9 ± 0.9 ^b^	3.1 ± 1.3 ^a^	1.4 ± 0.7	1.4 ± 0.7	1.2 ± 0.4	1.2 ± 0.4
Submucosa lymphoid cells	2.3 ± 1.1	2.3 ± 1.1	2.6 ± 1.1 ^b^	3.4 ± 0.7 ^a^	2.2 ± 0.9	2.9 ± 0.9	2.4 ± 1.0 ^b^	3.4 ± 0.7 ^a^
Submucosa granulocytes	2.5 ± 1.2 ^b^	3.5 ± 1.0 ^a^	2.2 ± 0.9 ^b^	3.6 ± 1.0 ^a^	1.9 ± 0.9	2.7 ± 0.7	2.1 ± 0.7 ^b^	2.8 ± 0.4 ^a^
Lamina propria lymphoid cells	1.9 ± 0.6	2.2 ± 1.1	2.5 ± 0.7 ^b^	3.4 ± 0.7 ^a^	2.8 ± 1.0	3.1 ± 1.0	2.4 ± 0.8	2.5 ± 1.1
Lamina propria granulocytes	2.4 ± 1.0	3.4 ± 1.1	2.2 ± 0.8	2.3 ± 1.3	2.1 ± 0.7	2.4 ± 0.5	1.5 ± 0.5	1.4 ± 0.5

CTRL – fish fed the control diet, Blend – fish fed the blend diet. Scores 1–9 were assigned to villi length/integrity, submucosa width, and lamina propria width; while scores 1–5 were assigned to denote the presence of leucocytes (i.e., lymphoid cells and granulocytes) in the submucosa and lamina propria. Values presented as mean ± SD (n = 10 samples per dietary treatment), based on a semi-quantitative analysis. Independent samples t-test was employed to detect significant differences between the groups within each intestinal section. Different letters denote significant differences between dietary treatments.

## Discussion

The production and economic viability of the aquaculture sector are adversely impacted by the rising incidence of disease outbreaks in fish farms^7^. Functional feeds are being recognised as promising tools to enhance fish robustness^10^, a key factor for boosting the profitability of the aquaculture sector. Gaining an in-depth understanding of the morphology and functional potential of the gastrointestinal tract is crucial for comprehending the functionality of novel feed components and allow comparison of results among studies. The intestinal health of farmed fish is intimately related to both nutrition and immunity^1^, both of which can affect the morphology and functions of the intestine. To advance the field of fish intestinal health, we conducted a comprehensive characterisation of the European seabass intestine and propose specific indices and detailed scoring system that were correlated with intestinal functionality.

In a previous study, Verdile et al.^5^ performed a detailed examination of the intestine of rainbow trout (*Oncorhynchus mykiss*), exploring different sections in specimens of varying weight ranges. Although growth-induced modifications of the intestine’s morphology were minimal, marked disparities were noted between the different sections of the intestine. Previous studies on European seabass intestine have only examined the anterior^25^, posterior/rectal segments^18^, or both the anterior and posterior segments^19^. In contrast, our study provides a comprehensive analysis of the four distinct sections of the European seabass intestine − anterior, mid, posterior, and rectum. The histomorphological parameters, gene expression patterns, and the position of the associated clusters in the PCA plot indicate the distinct morphology, functionality, and location of the different segments of the intestine. The samples collected from the anterior segment were linked to parameters associated with the processes of digestion and absorption. This segment exhibited the largest absorptive area and the longest villi, as well as the thinnest submucosa and highest count of neutral GC. In fish, the intestinal mucus layer produced by the GC is thought to play different roles, ranging from lubrication, absorption and immune defence^34^. The neutral GC, in specific, have been previously linked with improved digestion and absorption in European seabass^19^. The gene patterns observed in this segment further corroborate the importance of the anterior intestine within the digestive tract of fish. The feed efficiency of fish is highly dependent on both the digestive enzymes and the proteins involved in nutrient transportation processes^35^. In the present study, the involvement of anterior segment in the carbohydrates and lipid digestion and absorption is highlighted by the high expression of the genes encoding for the carbohydrate enzyme maltase (*malt*) and the fatty acid-binding protein 2 (*fabp2*) observed in this section. Therefore, the increased surface area observed in the anterior intestine in comparison with the other segments, coupled with the highest counts of neutral mucin-secreting cells, and high expression of genes that encode for digestive and transport proteins indicate that the region has the ideal environment for both nutrient digestion and absorption, in accordance with previous reports^26^.

The rectum and posterior intestine samples were found to be associated with immune-related variables. We observed the highest expression of immune-related genes in the rectum, followed, in general, by the posterior section. Moreover, the highest number of acid GC, that aid in protection against invading pathogens^36,37^, were found in the posterior/rectal sections, highlighting the importance of these segments for the intestinal immunity. A previous study examining in European seabass with a final body weight of 59 g revealed morphological differences between the posterior intestine and the rectum^18^. Our results show that the rectum of European seabass has longer villi compared to the posterior intestine and the posterior intestine has a higher density of GC, corroborating the findings of Torrecillas et al.^18^. In contrast to this study, we observed no variation in submucosa width between the posterior intestine and rectum, despite the latter having more lymphoid cells in the submucosa. These differences could be attributed to variations in fish body size (i.e., 60 vs 200 g in the present study) or exposure to different environmental challenges that might affect leucocyte infiltration. Our study compared four separate segments along the intestinal tract and revealed marked morphological differences in the rectum compared to the other three sections. Specifically, this region displayed the thickest muscularis, narrowest lamina propria, and longest microvilli. The thick muscularis layer observed in rectum suggests that intestinal motility^38^ is crucial in this section, potentially for the release of waste. In addition, the longer microvilli of the rectum indicate that some important absorption processes still occur in this segment. When examining the gene expression patterns, it appears that the rectum is primarily involved in water absorption, rather than nutrient digestion, as compared to the other segments. As one of the main osmoregulatory organs in marine teleost, the intestinal epithelium plays a key role in water transport-processes through the action of membrane proteins, such as aquaporins^39^. Similarly to findings reported by Giffard-Mena et al.^40^, the mid, posterior, and rectal segments of the intestine seem to be more relevant in the salt-water interactions across the intestinal barrier in European seabass, as evidenced by their high expression of *aqp1* compared to the anterior section.

The expression levels of the intestinal brush border membrane enzymes herein studied were segment-specific, except for alkaline phosphatase (*alp*). This enzyme displayed a consistent expression pattern across all the intestinal segments. The intestinal *alp* has been recognised for its role in preventing inflammation, both in the gut and at the systemic level^41^. According to the PCA plot, the variables *malt* and *fabp2* were found to be associated with the anterior section, along with other digestion/absorption-related parameters. In contrast, the expression of the *alp* gene appeared in the quadrants where the posterior and rectum samples clustered, in association with immune-related variables. Therefore, the ubiquitous expression of the *alp* gene throughout the intestine may play a role in maintaining intestinal homeostasis, which supports previous findings^42^.

The selected gene expression patterns were unable to differentiate between the mid and posterior regions. Nonetheless, several histomorphometric indices, such as the absorption area, microvilli height, number of GC and acid GC, area of GC, and quantity of leucocytes in the submucosa and lamina propria, were able to distinguish these regions. While the posterior intestine samples were positioned in the same quadrant of the PCA biplot as the rectum samples, the mid intestine samples clustered in a specific area of the PCA biplot, and without any clear association with the assessed variables. The mid intestine seems, therefore, to be an intermediate region for both absorption and immune-related processes.

In order to understand the turnover of the enterocytes in different sections of the intestine, we analysed the localization and distribution of the proliferating cell nuclear antigen (PCNA) by immunostaining positive cells. The anterior portion of the intestine, followed by the rectum, had the strongest PCNA signalling, although there were differences in localization. Similar to what was observed in rainbow trout^5^, the PCNA signal was detected both at the basis of the villi and at the villi branching points in the anterior intestine of European seabass, while in the rectum, PCNA immunolocalization was restricted to the base of the folds. The results suggest that the stem cell zone is most likely located at the basal part of the villi. This is consistent with previous findings in rainbow trout by Verdile et al.^43^, who found that the *sox9*^+^ cell zone (a marker for stem cells) in the intestine was similar to the PCNA^+^ cell zone. The understanding of the stem-cell population’s localization in the gastrointestinal tract is crucial for the development of novel in vitro tools, such as organoids, for the study of fish intestine. Organoids have recently emerged as one of the most promising tools for studying the intestinal health of farmed animals^44^.

Fish nutrition studies should accurately report the impacts of feeds on the intestinal health, and for this it is important to precisely describe the changes in the intestine segment selected for the analysis. Based on our findings, we propose that the intestine of European seabass can be divided into four different segments with unique morphological and/or gene expression profiles: 1) the anterior intestine, 2) the mid intestine, 3) the posterior intestine, and 4) the rectum. It is important to note that the length of the intestine can vary with fish size, and therefore, we recommend a standardized method for identifying and sampling these segments. Specifically, the anterior intestine should be sampled immediately after the pyloric ceca; the mid intestine should be sampled from the middle region between the pyloric ceca and the ileorectal valve, the posterior intestine should be the portion preceding the ileorectal valve, and the rectum should be the segment immediately after the ileorectal valve. Using this standardized approach for sample collection and nomenclature for intestine segments will ensure consistent and accurate reporting of feed impacts on intestinal health and allow comparison with future studies.

After conducting a detailed study on the different intestinal segments of European seabass, we aimed to investigate the effects of a novel macro- and microalgae blend on the intestinal functionality of the fish. Although the dietary inclusion of the blend did not affect fish growth performance or feed utilization parameters, the results suggest that the diet can alter the intestine morphology. Most studies that evaluated fish feed utilization and immune status relied either on the anterior intestinal segment^19^ or on the posterior/rectal segment^18^, respectively. However, in this study, the subtle differences between the CTRL and the Blend diet were mostly perceived in the mid intestine. When the impact of the blend was evaluated in the different intestinal segments using either a quantitative or semi-quantitative approach, the mid section presented, in general, the highest response towards the diet, which may indicate that this segment is more sensitive to external stimuli. The feeding trial lasted for only 8 weeks, hence the observed villi shortening and the reduced number of acid GC in the mid intestine are likely to be an adaptive response to the diet. Further evaluation of such alterations in the intestine structure is required after a longer-term feeding trial. Nonetheless, some immunomodulatory effects were already observed in various intestinal sections, including thickening of the submucosa (mid intestine), widening of the lamina propria (anterior and mid sections), an increase in the number of lymphoid cells in the submucosa (mid and rectum segments), granulocytes in the submucosa (all sections), and lymphoid cells in the lamina propria of the mid intestine. These observations, along with the increased expression of immune- and oxidative stress-related genes (*il-8*, *pisc1* and *gpx*), in the mid region, point towards an immunomodulatory effect promoted by the algae blend. This potential functionality should be further explored after a disease challenge, such as a bacterial infection. However, it is important to note that experimental conditions, namely temperature and salinity, can influence gut structure and functionality and yield varying outcomes^45–47^. In a previous study, we observed that European seabass fed a micro- and macroalgae blend and reared under similar temperature conditions as the present trial (21 °C), but with higher salinity levels (35‰), had an enhanced mucosal immune response upon infection with *Tenacibaculum maritium*^17^. Overall, these results suggest that dietary supplementation with algae could be a promising strategy to improve the robustness of European seabass.

To evaluate the impact of feeds on fish intestinal health, researchers have often conducted a semi-quantitative/score-based analysis focused on a determined intestinal segment. Such scoring system commonly used in literature often lacks clear definition and description of the selected indices and can lead to unreproducible results^48,49^. Moreover, due to the lack of a well-defined and validated scoring system, it is more likely for untrained individuals to make errors and have unreproducible results^20^. As morphologic traits vary throughout the tract, it is crucial to assess each section's specific morphologic traits and all samples beforehand to ensure the accurate assignment of the score. Hence, our study provides a detailed description of a set of scores for the semi-quantitative assessment of several morphological parameters of the intestine such as 1) villi length and integrity; 2) submucosa thickness; 3) lamina propria width; 4) submucosa lymphoid cells; 5) submucosa granulocytes; 6) lamina propria lymphoid cells; and 7) lamina propria granulocytes. The proposed scoring system includes validated and clearly defined criteria for each intestinal section, allowing researchers to select a scoring method that generates reliable and consistent data. Alternatively, recent studies investigating the morphology of the intestine have utilized quantitative approaches to produce precise measurements^18–20^. In the present work, we have also developed a detailed description of the indices and methods used for the quantitative assessment, which also allow direct comparisons among intestinal segments. We have used an image analysis software to automatically estimate the absorptive area, count the GC, and evaluate cellular proliferation (determined by the total area occupied by PCNA^+^ cells). For all other parameters, we have employed a semi-automatic approach. The quantitative assessment method enables us to measure the same parameters that are commonly evaluated through a score-based analysis (e.g., villi length, submucosa and lamina propria widths, and leucocyte infiltration)^20,48–50^, as well as several other additional parameters such as total absorptive area, muscularis thickness, and total GC counts in a cross-section. This approach has enormous potential to further evolve into a completely reliable automatic image analysis using artificial intelligence technologies for fast-track evaluation of fish intestinal health.

Although the semi-quantitative analysis is a less time-consuming and relatively easier method to assess the intestine histomorphology, it relies on pre-defined scores, which can increase bias^51^. In addition, unlike the quantitative assessment, the semi-quantitative analysis requires highly trained and experienced observers^20^. In the present study, we found a significant correlation between the values obtained by both quantitative and semi-quantitative analyses for the parameters evaluated within each intestinal segment. This indicates that both methods are valid for assessing intestinal health. While the semi-quantitative analysis may be useful for a quick pre-assessment, it is important to define the scoring system correctly to ensure good repeatability. However, we prefer the quantitative approach overall, as it allows for more easily comparable measurements across studies. To ensure robust and reproducible data from both methods, it is crucial that studies provide a detailed description of the methodology for each index in the methodology section.

In conclusion, by providing an in-depth characterisation of the different segments of the intestine (i.e., anterior, mid, posterior, and rectal) of European seabass, the present study provides valuable information about the intestinal morphology. Overall, we found that histological characteristics and gene expression profiles were specific to each intestinal segment. The anterior region appears to be primarily associated with absorption processes, with the largest absorptive area, longest villi, and a high expression of genes encoding digestive and transport proteins. In contrast, the rectum, followed by the posterior intestine, displayed the highest expression values for immune-related genes, highlighting their importance for mucosal immunity. The thick muscularis and long microvilli observed in rectum, coupled the high expression of the *aqp1* gene, most likely indicates that both waste release and water absorption are also important processes occurring in this segment. Finally, the mid intestine appears to be a transitional region for both absorption and immunity. Hence, the generated knowledge can assist in arriving at better decisions regarding the choice of the appropriate segment and morphometric indices for future experiments to study nutritional modulation and its effects on intestinal health. Furthermore, transition from a semi-quantitative to a quantitative analysis of intestine histomorphology is crucial for adopting digital technologies that produce more precise and fast histology-based results. As a futuristic perspective, the quantitative analysis offers a promising avenue for machine learning approaches in this field, allowing for high throughput image analysis of intestinal histological sections using artificial intelligence.

## Supplementary Information

Supplementary Information.

## Data Availability

Data will be made available, from the corresponding author, upon reasonable request.
