# Supplementary material for: An in-depth characterisation of European seabass intestinal segments for assessing the impact of an algae-based functional diet on intestinal health
Source: Sci Rep. 2023 Jul 19;13:11686. doi: 10.1038/s41598-023-38826-y (PMC10356848; doi:10.1038/s41598-023-38826-y)
Supplement: Supplementary file 1 — Supplementary Information. [file 41598_2023_38826_MOESM1_ESM.docx]

**Supplementary Table S1. Description of the parameters used to evaluate the intestinal morphology of European seabass juveniles – quantitative analysis.**

| **Parameter** | **Description** |
| --- | --- |
| **Cross-sectional perimeter (mm)** | The cross-sectional perimeter was automatically determined by the software after encircling the cross section using the commands “Measure” -> “Measurement and ROI” -> “Arbitrary line”. |
| **Absorption area (mm²)** | The total area occupied by the villi – absorption area - was determined automatically by the software. Firstly, a polygon was drawn from the base of the villi, right after the submucosa, using the commands “Automatically create ROI” -> “Closed Polygon”. Then, the software automatically determined the area occupied by the villi based on color, using the following commands: “Measure” -> “Count and Measure” -> “Manual HSV Threshold” -> “New Threshold” (select the tissue area with the tool) -> “Count and Measure”. |
| **Muscularis thickness (μm)** | The muscularis was measured from the outer layer of the section until the outer layer of the submucosa, in eight regions along the section, using the following commands: “Measure” -> “Measurement and ROI” -> “Arbitrary line”. The average muscularis thickness per section was determined. |
| **Submucosa width (μm)** | The submucosa width was measured from the outer to the inside limits of the submucosa layer, in eight regions along the section, using the commands “Measure” -> “Measurement and ROI” -> “Arbitrary line”. The average submucosa width per section was determined. |
| **Lamina propria width (μm)** | In eight villi selected for the villi length measurement, three measurements of the lamina propria width were made along the villi (one at the base, one in the middle and one at apex of the villi), using the commands “Measure” -> “Measurement and ROI” -> “Arbitrary line”. The average lamina propria width per section was determined. |
| **Villi length (μm)** | The eight highest villi per section were selected for the measurement. The villi length was measured from the tip of the villi to the apex, using the following commands: “Measure” -> “Measurement and ROI” -> “Polyline”. The average villi length of per section was determined. |
| **Microvilli height (μm)** | In the eight villi selected for the villi length measurement, eight measurements of the microvilli heigh were made along the villi, using the commands “Measure” -> “Measurement and ROI” -> “Arbitrary line”. The average microvilli height per section was determined. |
| **Acid GC** | Based on the color of the cells (blue), the number of acid GC was automatically determined by the software, using the following commands: “Measure” -> “Count and Measure” -> “Manual HSV Threshold” -> “New Threshold” (select the blue cells with the tool) -> “Count and Measure”. |
| **Neutral GC** | Based on the color of the cells (magenta), the number of neutral GC was automatically determined by the software, using the following commands: “Measure” -> “Count and Measure” -> “Manual HSV Threshold” -> “New Threshold” (select the magenta cells with the tool) -> “Count and Measure”. |
| **Goblet cells (GC)** | Sum of the acid GC and neutral GC. |
| **Average area GC (μm²)** | After automatic counting of the GC, the average area of the cells is provided by the software. |
| **Submucosa lymphoid cells** | Six photos of the submucosa were randomly taken at 400 x magnification. All lymphoid cells present in those photos were counted and an average number of lymphoid cells per section was determined. |
| **Submucosa granulocytes** | Six photos of the submucosa were randomly taken at 400 x magnification. All granulocytes present in those photos were counted and an average number of granulocytes per section was determined. |
| **Lamina propria lymphoid cells** | Six photos of the lamina propria were randomly taken at 400 x magnification. All lymphoid cells present in those photos were counted and an average number of lymphoid cells per section was determined. |
| **Lamina propria granulocytes** | Six photos of the lamina propria were randomly taken at 400 x magnification. All granulocytes present in those photos were counted and an average number of granulocytes per section was determined. |
| **PCNA^+^ cells total area** | The PCNA^+^ cells (immunohistochemistry) were automatically detected by the software based on their color (brown), using the following commands: “Measure” -> “Count and Measure” -> “Manual HSV Threshold” -> “New Threshold” (select the brown cells with the tool) -> “Count and Measure”. |

All measurements were performed using the imaging software Olympus cellSens Standard 2.2.

**Supplementary Table S2. Primers used in the RT-PCR analysis and corresponding annealing temperature (°C), efficiency of the RT-PCR (%), accession number (GenBank ID) and reference.**

| **Gene** | **Primer Forward** | **Primer Reverse** | **Annealing T (°C)** | **Efficiency (%)** | **GenBank ID** | **Reference** |
| --- | --- | --- | --- | --- | --- | --- |
| ***18s*** | CCAACGAGCTGCTGACC | CCGTTACCCGTGGTCC | 58 | 100 | AY831388.1 | Nuñez-Ortiz et al.^52^ |
| ***β-actin*** | CAAAGCCAACAGGGAGAAGATGA | ACCGGAGTCCATGACGATAC | 60 | 102 | AJ537421 | Couto et al.^53^ |
| ***ef1α*** | AACTTCAACGCCCAGGTCAT | CTTCTTGCCAGAACGACGGT | 58 | 101 | AJ866727.1 | Azeredo et al.^54^ |
| ***gapdh*** | CAAGGTTGAGGGTGACAAGC | CAGCCTCACCCCATTTGATG | 60 | 102 | AY863148 | Couto et al.^53^ |
| ***igM*** | AGAGGAGCTGCAGGAGGACA | GGACCTCCAGGCTGTGTGAC | 60 | 105 | AJ400233 | Couto et al.^53^ |
| ***cd4*** | ACTTGTGATTTGGGCAGTCC | TGATGAGATGAGTGGGGTGA | 60 | 102 | AM849812 | Couto et al.^53^ |
| ***il-6*** | AGGCACAGAGAACACGTCAAA | AAAAGGGTCAGGGCTGTCG | 58 | 103 | AM490062.1 | Machado et al.^55^ |
| ***il-8*** | CGCTGCATCCAAACAGAGAGCAAAC | TCGGGGTCCAGGCAAACCTCTT | 58 | 100 | AM490063 | Machado et al.^56^ |
| ***il-1β*** | AGCGACATGGTGCGATTTCT | CTCCTCTGCTGTGCTGATGT | 58 | 94 | AJ269472 | Azeredo et al.^57^ |
| ***tnf-α*** | AGCCACAGGATCTGGAGCTA | GTCCGCTTCTGTAGCTGTCC | 60 | 107 | DQ070246 | Machado et al.^56^ |
| ***tcrβ*** | GACGGACGAAGCTGCCCA | TGGCAGCCTGTGTGATCTTCA | 58 | 106 | AJ493441 | Torrecillas et al.^58^ |
| ***tlr9*** | TCTTGGTTTGCCGACTTCTTGCGT | TACTGTTGCCCTGTTGGGACTCTGG | 60 | 107 | KX399289 | Byadgi et al.^59^ |
| ***casp3*** | TGATGTCGTCTCTGCCGTAG | ACCACCTCATACGCATCCTC | 60 | 104 | DQ345773 | Couto et al.^53^ |
| ***pcna*** | TGAAGTGTGCAGGAAACGAAGA | GGCGAGTGTGTCTGCATTGT | 60 | 106 | JQ755266 | Rawling et al.^60^ |
| ***pisc1*** | TCGTCCTCATGGCTGAACC | GCGGTTAAAGCGCTGATATTG | 60 | 105 | MT066191 | Barroso et al.^61^ |
| ***gpx*** | GTTTGGACATCAGGAGAACTGC | CATCGCTGGGGTATGGAAGC | 58 | 102 | DT044993 | Azeredo et al.^54^ |
| ***sod*** | GGAGAGTGATTCAGCCCCTG | GGAAACCATGCTCACCAGGA | 58 | 102 | CX660893.1 | Machado et al.^55^ |
| ***cat*** | GAGGTTTGCCTGATGGCTAC | TGCAGTAGAAACGCTCACA | 60 | 108 | FJ860003 | Morcillo et al.^62^ |
| ***alp*** | TTACCTCTGTGGGGTCAAGG | TAGCCCATTTGAGGATGGAG | 60 | 94 | FJ860000 | Couto et al.^53^ |
| ***malt*** | ATGCCATGGAGGTGACTTTC | CAACCATTTCAGGCGTAGGT | 60 | 105 | AM419039 | Couto et al.^53^ |
| ***fabp2*** | CCGCAACGACAACTATGATAAG | TGGACTCTTTGATGTGAAACTTG | 60 | 106 | MF566099 | Kaitetzidou et al.^63^ |
| ***aqp1*** | CAAGGCAGTCATGTATATTG | AGAGAGTTGAGCCCCAGT | 60 | 105 | DQ924529 | Giffard-Mena et al.^64^ |

*18s*, 18S rRNA; *β-actin*, Beta actin; *ef1α*, Elongation factor 1 alpha; *gapdh*, Glyceraldehyde-3-phosphate dehydrogenase; *igM*, Immunoglobulin M; *cd4*, Cluster of differentiation 4; *il-6*, Interleukin-6; *il-8*, Interleukin-8; *il-1β*, Interleukin-1 beta; *tnf-α*, Tumor necrosis factor-alpha; *tcrβ*, T-cell receptor antigen receptor beta chain; *tlr9*, Toll-like receptor 9; *casp3*, Caspase 3; *pcna*, Proliferating cell nuclear antigen; *pisc1*, Piscidine 1; *alp*, Alkaline phosphatase; *malt*, Maltase; *fabp2*, Intestinal fatty acid binding protein; *aqp1*, Aquaporin 1; *gpx*, Glutathione peroxidase; *sod*, Superoxide dismutase; *cat*, Catalase.

**Supplementary Table S3. Histomorphology of the different intestine sections of European seabass using a semi-quantitative analysis.**

|  | **Intestine Sections** | | | |
| --- | --- | --- | --- | --- |
|  | **Anterior** | **Mid** | **Posterior** | **Rectum** |
| Villi length and integrity | 2.4 ± 1.8 | 3.0 ± 2.2 | 2.6 ± 1.2 | 1.8 ± 1.1 |
| Submucosa width | 1.7 ± 1.0 | 3.0 ± 1.8 | 2.6 ± 1.1 | 2.0 ± 0.7 |
| Lamina propria width | 2.6 ± 1.7 | 2.5 ± 1.2 | 1.4 ± 0.7 | 1.2 ± 0.4 |
| Submucosa lymphoid cells | 2.3 ± 1.0 | 3.0 ± 1.0 | 2.5 ± 0.9 | 2.8 ± 1.0 |
| Submucosa granulocytes | 2.8 ± 1.2 | 2.7 ± 1.2 | 2.3 ± 0.9 | 2.5 ± 0.7 |
| Lamina propria lymphoid cells | 2.1 ± 0.0 | 2.5 ± 0.8 | 2.5 ± 1.0 | 2.0 ± 0.9 |
| Lamina propria granulocytes | 2.5 ± 1.1 | 2.3 ± 1.1 | 2.3 ± 0.6 | 1.5 ± 0.5 |

Scores 1-9 were assigned to villi length/integrity, submucosa width, and lamina propria width; while scores 1-5 were assigned to denote the presence of leucocytes (i.e., lymphoid cells and granulocytes) in the submucosa and lamina propria. Values presented as mean ± SD (n = 20 samples per section), based on a semi-quantitative analysis.

**
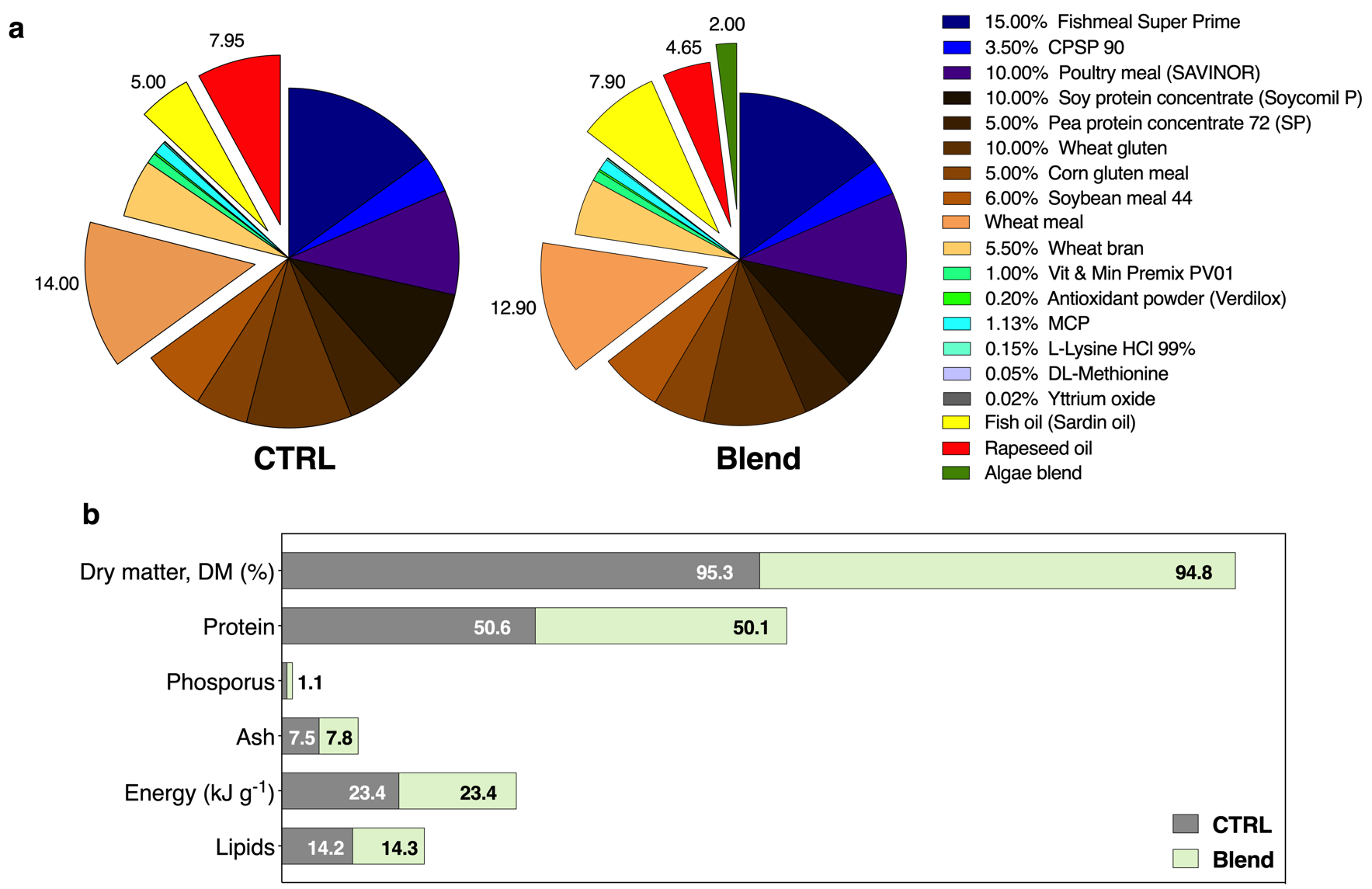
**

**Supplementary Figure S1. Formulation and proximal composition of the experimental diets.** Ingredients in % (a) and proximate composition in % DM (B) of the experimental diets.

**
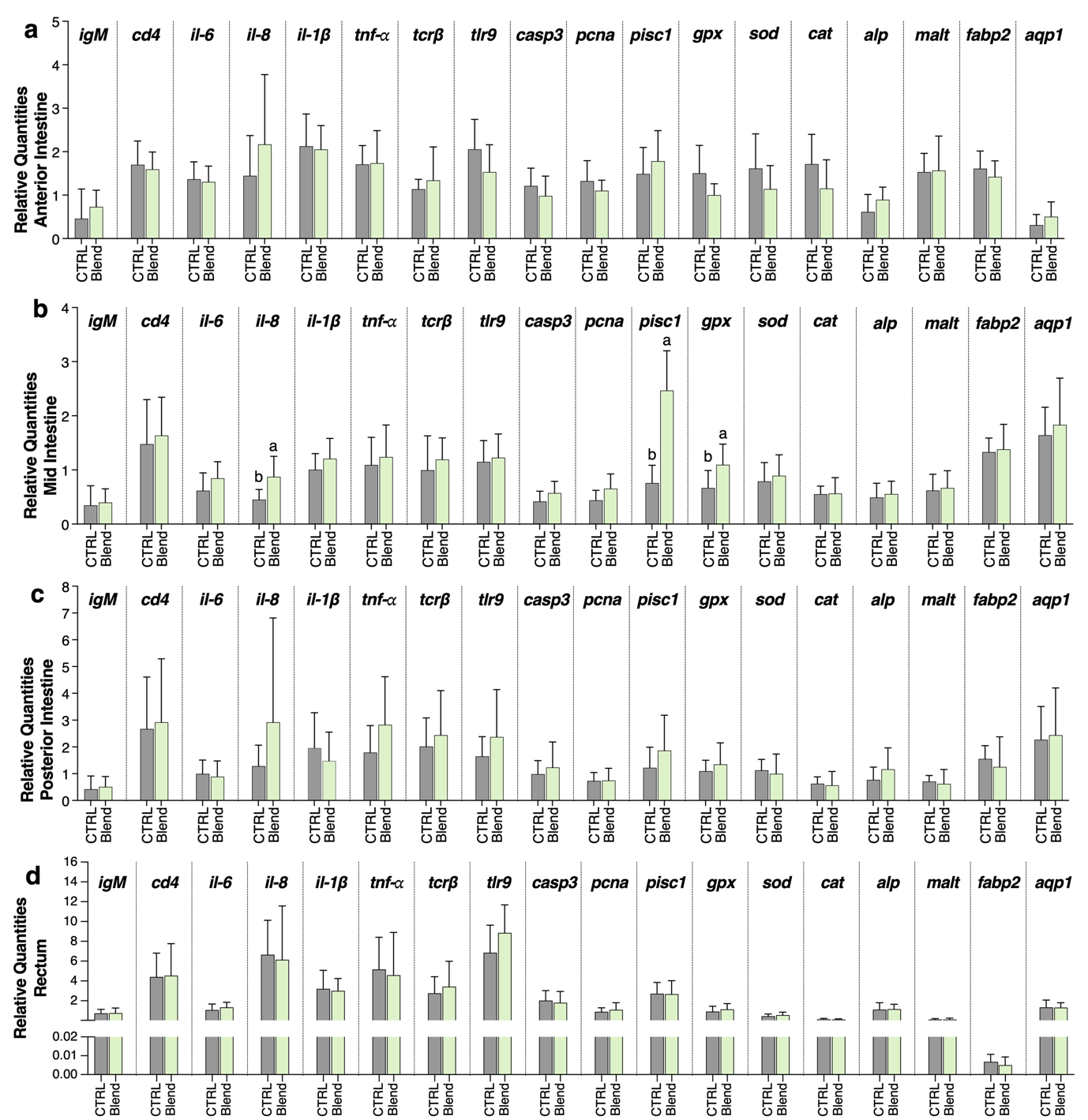
**

**Supplementary Figure S2.** **Genes expression in the different sections of the intestine of European seabass juveniles fed the control or the blend diets.** a) Anterior intestine; b) Mid intestine; c) Posterior intestine; and c) Rectum. Relative quantities of immune-related genes (igM, cd4, il-6, il-8, il-1β, tnf-α, tcrβ, tlr9, casp3, pcna and pisc1); genes associated with oxidative stress (gpx, sod, and cat); and genes related to nutrient digestion and absorption (alp, malt, fabp2, and aqp1) are presented as mean ± SD (n = 16 samples per dietary treatment). Independent samples t-test was employed to understand the significant differences between the study groups. Significant differences are detected only for the mid intestine, with different letters denoting significant differences between dietary treatments.

**
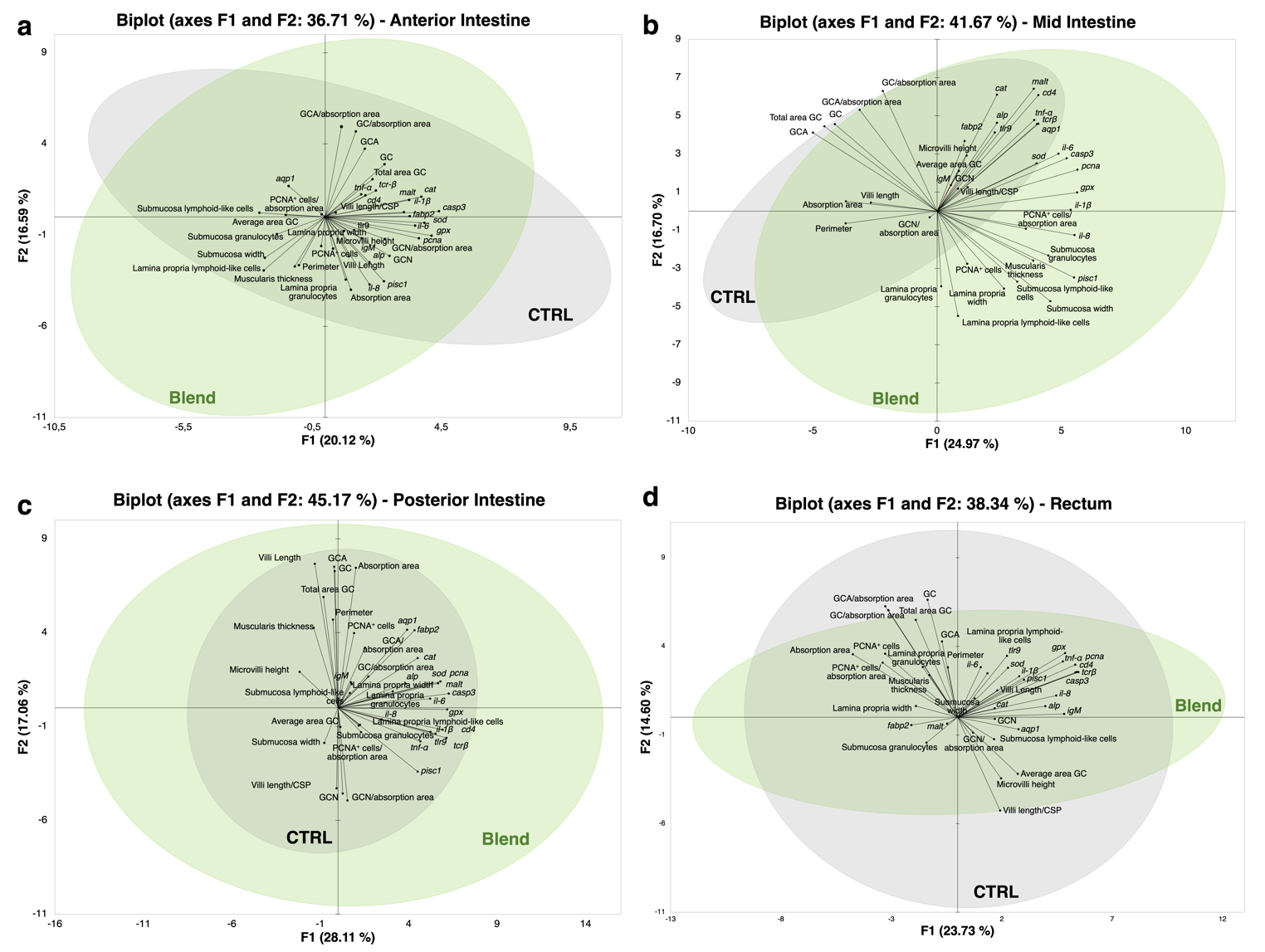
**

**Supplementary Figure S3.** **Principal component analysis (PCA) biplot for all variables analysed.** a) Anterior intestine; b) Mid intestine; c) Posterior intestine; and d) Rectum. Variables are displayed as loading vectors. Confidence ellipses (95%) are drawn for each dietary treatment: CTRL – grey; Blend – green.


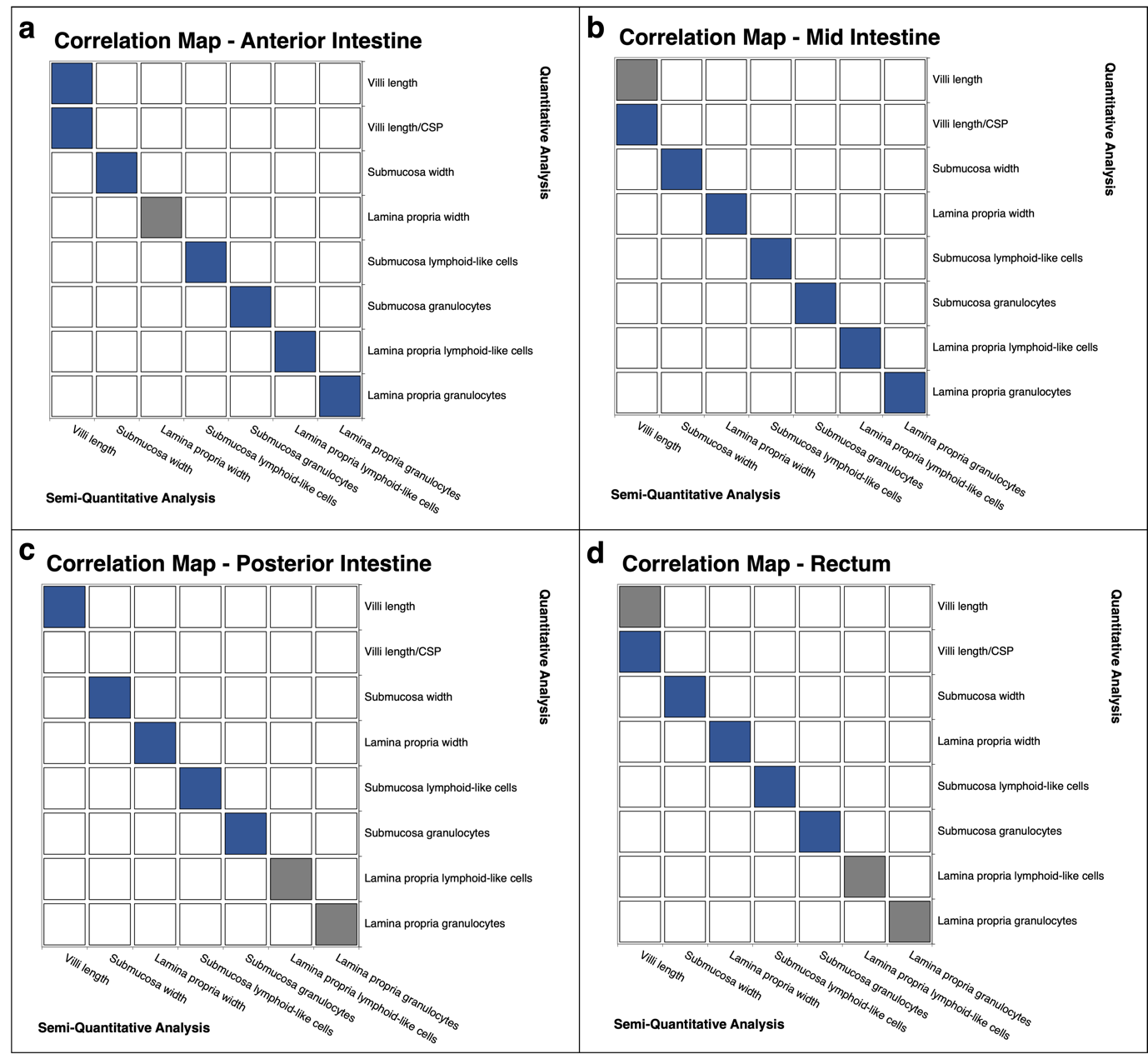


**Supplementary Figure S4.** **Correlogram showing the correlation between the equivalent histomorphometric variables measured by quantitative and semi-quantitative analyses of the in the anterior (a), mid (b), posterior (c), and rectal (d) sections of the intestine of European seabass.** Correlations with statistical significance at P < 0.05 are displayed as blue and with statistical significance at P < 0.1 are displayed as grey.
